# The Home Learning Environment of Primary School Children with Down Syndrome and Those with Williams Syndrome

**DOI:** 10.3390/brainsci11060733

**Published:** 2021-05-31

**Authors:** Erica Ranzato, Andrew Tolmie, Jo Van Herwegen

**Affiliations:** Department of Psychology and Human Development, UCL Institute of Education, 20 Bedford Way, Bloomsbury, London WC1H 0AL, UK; andrew.tolmie@ucl.ac.uk (A.T.); j.vanherwegen@ucl.ac.uk (J.V.H.)

**Keywords:** Down syndrome, Williams syndrome, home learning environment, home numeracy environment, home literacy environment

## Abstract

Background and aims: Research on typically developing (TD) populations has shown that the home learning environment plays a significant role in cognitive development and learning, but very little is known about the home learning environment of children with Down syndrome (DS) or children with Williams syndrome (WS). The present study examined and compared, for the first time, the home learning environment of children diagnosed with DS and children diagnosed with WS to investigate whether different cognitive profiles were reflected in their home literacy and number experiences. Methods and procedures: Quantitative and qualitative data were collected through a web-based survey from 58 parents and one foster parent of primary school children with DS (n = 35) and WS (n = 24) mostly based in the UK. The survey targeted the children’s general level of functioning and academic skills; type, format, and frequency of home learning activities; parents’ expectations for their child’s academic outcomes; parents’ attitudes towards literacy and mathematics; children’s interest towards mathematics; and the use of technology to support home learning activities. Outcomes and results: Our results showed that, overall, the home learning environment of children with DS and children with WS were similar but changed based on the child’s cognitive profile. Comparative analyses showed that parents of children with DS engaged more often in activities supporting counting than parents of children with WS, despite both groups reporting difficulties with this skill. Moreover, our results indicated that literacy-based activities occurred more often than mathematics-based activities and that the home numeracy environment was characterized by activities supporting different mathematical skills such as counting, arithmetic, and numeracy. Parents in both groups engaged with their child in both formal and informal literacy and mathematics-based activities, but informal activities occurred more often when supporting counting and number recognition skills. Conclusions and implications: The current study provides evidence that the home learning environment of children with DS and children with WS include different literacy- and mathematics-based activities and that the home learning environment changes on the basis of the child’s strengths and weaknesses. The findings are discussed in relation to previous studies and the impact on parental interventions.

## 1. Introduction

Down syndrome (DS) and Williams syndrome (WS) are two genetic developmental disorders that have similar overall cognitive impairments within the mild-to-moderate learning difficulties range but are characterized by differently uneven cognitive profiles; individuals with DS show poorer language expressive abilities and short-term memory skills as compared to their visuospatial skills [1], whereas individuals with WS have better language and short-term memory abilities as compared to their visuospatial skills [2].

Cross-syndrome studies comparing WS and DS populations have investigated how these specific cognitive phenotypes influence later cognitive development as well as the alternative developmental trajectories that can emerge from compensatory mechanisms [3]. For example, a study by Steele et al. [4] assessed reading abilities in children with DS and in children with WS and reported that the two groups differed in how they developed early reading; children with DS performed poorly on rhyme matching, phoneme matching, and receptive vocabulary as compared to children with WS who performed relatively well on all measures assessing reading precursors. A study by Varuzza et al. [5] examined different aspects of writing abilities in individuals with DS (mean CA = 19.5) and individuals with WS (mean CA = 19.7). The authors found that only the DS group made more errors than the control group in the word dictation task and they explained this finding as a consequence of difficulties in employing orthographic and lexical knowledge. Finally, Van Herwegen et al. [6] examined the development of mathematical abilities in individuals with WS (mean CA = 19.4) and in individuals with DS (mean CA = 21.9) and suggested that, although their mathematical performances were similar, they were driven by different developmental pathways, i.e., mathematical abilities were related to visuospatial abilities for the DS group and to the non-symbolic number abilities for the WS group. 

The neuroconstructivist approach proposes a framework for the study of cognitive development characterized by a dynamic multi-level approach [7] in which genes, brain, behaviour, and environment interact multi-directionally throughout development [8]. Following the neuroconstructivist approach, in the present study, we examined and compared the home learning environment of children diagnosed with DS and children diagnosed with WS to investigate whether different cognitive profiles and developmental pathways were reflected in the home literacy and number experiences. Given the different cognitive profile of these populations, this comparison study investigated the interaction between cognitive and environmental factors.

### 1.1. Home Learning Environment

The home learning environment refers to all of the activities and opportunities provided by a parent to support their child’s overall academic success [9]. It is a multifaceted construct that encompasses the frequency of home learning experiences, the availability of resources that promote learning, a child’s participation in the learning activities, and parental attitudes towards learning [10]. Cross-cultural research on typically developing (TD) populations suggests that the home learning environment during early years plays a pivotal role in the development of a child’s literacy skills [11] and mathematical abilities [12]. Parental expectations, beliefs, attitudes, and demographic characteristics have been shown to have an impact on early child academic development [13]. Furthermore, findings have revealed that parents who provide their child with a more stimulating and varied home learning environment during the early years were also more likely to continue to provide stimulating home learning environments when their child was older [10]. 

The home learning environment can be differentiated into home literacy environment and home numeracy environment. 

The home literacy environment (HLE) is often defined as the frequency of literacy-related activities in the home, such as shared parent–child book reading. However, the age of onset of parent–child book reading, the number of books in the home, the frequency of trips to the library, and parental attitudes such as the enjoyment of reading and beliefs about reading are also considered to be important aspects of the HLE [14]. Senechal et al. [15] suggested that children at home can be exposed to both formal literacy instruction activities, i.e., those activities where the attention is on the print itself, and informal literacy experiences, i.e., those activities where the print is present but is not the focus of the parent–child interaction. The existing studies in TD populations on HLE suggest that children’s exposure to books, in both formal and informal activities, is related to the development of vocabulary and listening comprehension skills and that parental involvement in teaching children about reading and writing words is related to the development of early literacy skills [11].

The concept of home numeracy environment (HNE) has been defined as any parent–child interactions with numerical content and has been operationalised as the frequency of mathematics-based activities that occur at home or the frequency of number talks observed during parent–child interactions [16]. LeFevre et al. [17] suggested that home number-based activities can also be divided into formal and informal activities. On the one hand, formal activities explicitly focus on mathematical abilities and are used by parents for the specific purpose of developing mathematical skills, such as practicing number names or simple sums. On the other hand, informal activities consist of real-world tasks during which parents’ teaching happens without an explicit purpose and the acquisition of number skills is likely to be incidental, such as playing card or board games that involve numbers, talking about money, and reading clocks.

Studies in TD populations have suggested that home numeracy activities occur less frequently than literacy activities [17,18]. Furthermore, findings from a qualitative study by Cahoon et al. [19] on parents’ experiences in relation to mathematical practices at home reported that, while reading was a structured daily activity that parents dedicated specific time to, number-related experiences were unstructured and did not occur at a prescribed time. A study by Blevins-Knabe, Austin, Musun, Eddy and Jones [18] compared the frequency of literacy-based and numeracy-based activities and reported that the frequency with which parents involved 4- to 6-year-old children in mathematical activities was related to their frequency of engaging children in literacy-based activities. Moreover, this study showed that the frequency with which parents involved their child in mathematical activities was related to their own attitudes towards mathematics, in that, those parents who enjoyed mathematics offered mathematical activities more frequently. Several studies have shown that most parents reported counting objects to be the most frequent mathematics-based activity [12,18,20] and that very few activities focused on other aspects, as parents often failed to grasp opportunities to incorporate extra numeracy components into daily activities [21]. Finally, del Rio et al. [22] found that parents who had high numeracy expectations for their child also reported engaging more frequently in advanced numeracy activities at home.

### 1.2. Home Learning Environment of Children with Down Syndrome and Children with Williams Syndrome

A few studies have investigated literacy-based learning activities of preschool children with DS [23,24,25,26], children with DS [27,28,29], and adults with DS [30], while no studies have explored mathematics-based resources and activities of parents of children with DS. To the best of our knowledge, there are no studies that have investigated home learning environment of children with WS.

The results from studies examining the home learning environment of individuals with DS have shown that most parents placed a high value on supporting their child’s literacy development and that the majority of parents were involved in regular literacy interactions with their child [23,26], although more so for reading than for writing [28]. The HLE of children with DS was rich [25] and positive, with the majority of children introduced to books when they were one year old and the majority of the families with mixed SES reporting having more than 50 children’s books at home [23,28] as well as a wide range of writing materials [28,30]. Parents of children aged from 3 months to 6 years reported reading to or with their child on a daily basis and using reading instructional materials daily with their child, such as flash cards or magnetic letters [23]. Parents also reported additional ways in which they facilitated literacy development, including active teaching, language games, exposure through TV programmes and other electronic media, and library visits [28,29]. These studies also reported that children with DS had a positive interest in reading, regardless of their age [26].

Furthermore, Al Otaiba, Lewis, Whalon, Dyrlund and McKenzie [23] investigated the lifelong literacy goals that parents had for their preschool children and found that developing their child’s literacy was a high priority for parents who reported goals such as recognising their alphabet, reading for meaning, reading for pleasure, reading chapter books, and reading for job purposes. These findings were confirmed in a study by Ricci and Osipova [29], where parents of children aged between 3 and 13 years described reading as a key goal for their child.

Finally, Trenholm and Mirenda [30] reported on the frequency of the use of computers and calculators by individuals with DS at home and they highlighted that adolescents and adults engaged with technology more often and in ways that were more “functional” than school-based activities as compared with the younger participants in the sample. However, most of the studies that have investigated the use of technology in this population have focused on the descriptions of the related challenges (e.g., Feng et al. [31]) and on their application for therapy interventions, and very little is known about the use of technology to support home learning activities for individuals with DS. To the best of our knowledge, there are no studies investigating the use of technology in the home environment of individuals with WS.

In summary, there is a lack of studies investigating the home learning environment of children with DS or children with WS. Although the studies that have examined the HLE in the DS population have suggested that parents attempt to compensate for areas of relative weakness by offering a rich HLE; the lack of studies investigating the HNE and parental attitudes towards mathematics in these populations does not allow for an exhaustive exploration of the interaction between cognitive profiles and the home learning environment. It is important to expand our understanding of the home learning experiences of children with DS or WS in order to indicate ways in which parents can enhance the home learning environment and support their child’s development at home. Moreover, this investigation can provide important evidence to support the development of targeted interventions aimed at supporting learning in these populations.

### 1.3. The Current Study

The current study investigated the home learning environment of primary school children with DS and primary school children with WS using a parental web-based survey and, for the first time, explored the HNE of these populations. This work compared home learning experiences of children with DS and children with WS in order to determine whether literacy and mathematical skills were being targeted and to explore interactions between the children’s cognitive profiles and the home learning environment. In particular, this study compared the following themes for children with DS and children with WS:

1. Parents’ reports of their children’s general level of functioning and academic skills.
2. Type (literacy, numeracy), format (formal, informal), and frequency of activities that occur at home.
3. Parents’ expectations and concerns regarding their child’s academic outcomes.
4. Parents’ attitudes towards literacy and mathematics and children’s interests and attitudes towards mathematics.
5. Use of learning resources and technology to support home learning activities.

On the basis of the existing literature on the HLE of individuals with DS and on the HNE of TD populations, the following predictions were made:

1. In line with the population’s cognitive profile, parents of children with DS would give lower scores on the VABS-II expressive scale, and parents of both groups would report difficulties with mathematical skills for their children.
2. Similar to TD populations, literacy-based activities would be more frequent than mathematics-based activities. Within the mathematics-based activities, counting would be the most frequent activity.
3. Similar to parents’ expectations towards reading in DS population, parents’ expectations towards their child’s academic outcomes would be high, in that parents would expect their child to achieve the targets set by the English national curriculum [32].
4. Similar to TD populations, the frequency of mathematics-based activities would be related to a parent’s attitude towards mathematics. No predictions were made on a child’s level of interest and motivation towards mathematics-based activities, due to lack of literature.
5. When technology is used to support learning, the pattern observed in the type of learning activities occurring would be the same as the one observed when no technology is involved.

## 2. Methods

### 2.1. Participants

A total of 80 families with a child diagnosed with either DS or WS took part in this study. Among the 80 participants there were only 59 respondents (DS group n = 35, WS group n = 24) who completed the questionnaire in its entirety and were included in the final sample; 57 respondents (96%) were mothers, 1 respondent was a father (2%), and 1 respondent was a female foster parent (2%). Overall, most of the families were well educated, white, and based in the UK. See Table 1 for all descriptive characteristics of the participants. 

### 2.2. Measures

A new online survey was developed for this study that allowed the inclusion of online communities. This survey was created on the basis of previous studies examining HNE of preschool TD children [13,17,33] and of the English national curriculum [32]. The survey was piloted with two parents (one for each neurodevelopmental disorder) to assess if the respondents understood the questions and were able to complete the questionnaire easily. The pilot version was modified in line with their feedback. In particular, some questions were reworded, one question type was changed, and two new questions were included. Thus, the pilot data were not included in the final data sample. The final survey included 66 questions grouped into 7 sections and included both closed- and open-ended questions, as respondents were invited to make additional comments at the end of each section.

#### 2.2.1. Children’s Demographic Data

The first section of the survey asked about children’s gender, age, clinical diagnosis, language spoken at home, school setting, and whether they had received any additional support in the last year. Parents were asked to report their child’s school target grades in three different areas, i.e., mathematics, reading and writing. Moreover, for each area, parents were asked to rate their child’s specific abilities as compared with their child’s overall abilities (i.e., better, in line, or worse than their overall abilities).

#### 2.2.2. Frequency of Home Learning Activities

In the second section of the survey, parents were presented with a list of 36 home activities (see Appendix A) and were asked to rate on a 4-point Likert scale, ranging from never (0) to more than once a week (3), how often they engaged in these activities with their child. Parents could select “not age appropriate” if they considered their child was too old or too young for the presented item. On the basis of previous studies, a list of home activities and resources were identified and classified into the following six different areas: literacy-based activities (n = 6); everyday life activities (n = 6); activities related to domain-general abilities that support mathematical development (e.g., visuospatial abilities) (n = 6); and mathematics-based activities broken down into three kinds (number skills (n = 6), arithmetic skills (n = 6), and broader mathematical skills (n = 6)).

Literacy-based activities were included on the basis of previous findings that have shown that both home numeracy- and home literacy-related activities were associated with children’s mathematical skills [18]. Moreover, everyday activities were included in the survey because of previous findings that have indicated that such activities facilitated the academic performance of TD children. Unlike previous studies, mathematical skills, in this study, were operationalized in a broader way and included children’s number and arithmetic skills, as well as their number sense, functional mathematics, and geometry abilities, which were considered to be components of their mathematical abilities. Hence, specific mathematics-based activities included activities targeting number skills (i.e., counting and number recognition) (n = 6), arithmetic skills (n = 6), and broader mathematical skills (n = 6) such as number foundations and functional mathematics. 

In order to measure the frequency of home learning activities, the category frequency (CF) score was computed for each participant as the average score of the six items presented in each category. In the case of items reported as “not appropriate”, these were excluded from the computation. Where more than 3 items were reported as “not appropriate” in the same category, the category was coded as “not appropriate”. The CF scores ranged from 0 to 3, with a higher score indicating a home environment where learning activities occur more frequently. 

#### 2.2.3. Use of Technology 

The third section of the survey included questions about a child’s use of technology (e.g., tablets, computers, TV, and videogames), and specifically the frequency and the purpose of the use of technology at home. Parents were also asked to report 3 names of literacy and numeracy applications and to report how often their child was using various mathematical applications (e.g., additions and subtractions games). Finally, parents were asked to report their concerns, if any, about their child’s use of technology. 

#### 2.2.4. Parents’ Expectations 

The fourth section of the survey included questions that asked about parents’ expectations. Parents were asked to indicate on a 10-point Likert scale (from not at all (0) to very well (10)) how well they expected that their child would master specific competencies at the end of primary school (e.g., counting up to 100). The 56 competencies listed in this section were linked to the home activities included in the second section of the survey and replicated the same classification, targeting the following: counting and number recognition skills (n = 8), arithmetic skills (n = 8), broader mathematical skills (n = 8), literacy skills (n = 8), skills related to domain-general abilities that support mathematical development (n = 8), and everyday life skills (n = 8). In order to validate participants’ responses, we included control items (n = 8) including mathematical skills included in the English national curriculum Year 5 and Year 6 (e.g., “converting between miles and kilometres”) and everyday life skills characterized by high levels of independence (e.g., using public transports independently). In order to measure parents’ expectations, the average score of the 8 items presented in each category was computed for each participant, i.e., the expectation score (ES). The ES ranged from 0 to 10, with a higher score indicating higher parental expectations. 

#### 2.2.5. Children’s and Parents’ Attitudes towards Numeracy and Literacy

In the fifth section of the survey, parents were asked to rate, on a 5-point Likert scale ranging from strongly disagree (1) to strongly agree (5), the extent to which they agreed with 6 statements about their own attitudes towards mathematics (e.g., “I like mathematics”) and literacy (e.g., “reading is important”), as well as 5 statements about their child’s attitudes towards numeracy (e.g., “my child enjoys mathematics”). When rating their child’s attitudes, a parent could select “not appropriate” if they considered that the statement was not appropriate for the stage of development of their child. For each participant, the parent’s attitude (PA) and the child’s attitude (ChA) scores were calculated as the average score of the items presented. Both PA and ChA ranged from 1 to 5, with a higher score indicating more positive attitudes.

#### 2.2.6. Vineland Adaptive Behaviour Scale II

The sixth section of the survey included items from the following five subdomains of the Vineland Adaptive Behaviour Scale II (VABS-II, [34]): receptive (13 items), expressive (15 items), written (13 items), living in the community (22 items), and fine motor skills (26 items). The VABS-II measures the general level of functioning in individuals from birth to 18 years and has been used in previous studies with children with DS [27]. Participants were asked to score their child’s behaviour on a scale from 0 (child never performs the behaviour or never performs it independently) to 2 (child usually performs the behaviours independently). For each participant, the total raw score for each subdomain was computed, with higher scores indicating more adaptive behaviours. 

#### 2.2.7. Participants’ Demographic Data

The final section of the survey asked the participants to provide their demographic information. The questions included participants’ ethnicity, country, and highest level of both education and mathematical education completed. We chose to use parental education as a proxy measure for socioeconomic status, as parental education is the most commonly used indicator of SES in research with children and adolescents and it is highly predictive of other variables such as income and occupation [35].

### 2.3. Procedure

Participants completed the online survey between December 2018 and May 2019 through an anonymous link that was made available through an online survey platform, Qualtrics. Participants were recruited through the Williams Syndrome Foundation UK, Down syndrome support groups across the UK, and through social media using methods such as the placements of announcements on relevant Facebook groups and Twitter. Participants could complete the questionnaire partially and could stop at any point or omit any question. They were requested to complete the survey using the same link within 2 weeks. All participants were informed about the content and scope of the study and gave written informed consent before starting the online survey. This project was reviewed according to procedures specified by Kingston University London and allowed to proceed (approval n. 1718CHA12). 

### 2.4. Data Analysis

In order to describe the sample, descriptive statistics for the VABS-II and the chronological age were presented. Then, a series of Mann–Whitney U tests were conducted in order to examine differences in the cognitive profile of children with DS and children with WS, as reported by parents. Fisher’s exact test was run to examine the significance of the association between child diagnosis and the type of school setting.

In order to examine the home learning environment, descriptive statistics for the CF, ES, PA, and ChA were presented. Then, a series of Mann–Whitney U tests were conducted in order to examine differences between children with DS and children with WS in the features of home learning environment. Friedman tests were run to determine if there were differences in the CF and ES scores between different categories. Pairwise comparisons were performed running separate Wilcoxon signed-rank tests with a Bonferroni correction for multiple comparisons.

Spearman’s correlations were run in order to determine whether there was an association between the cognitive and environmental factors that influence cognitive development and to determine whether there was an association between the different variables characterizing the home learning environment. 

Non-parametric analyses were conducted because of violation of the normality assumption and, in the case of the Mann–Whitney U tests, because of the small sample size of the WS group (n = 24). 

Qualitative data included comments provided by 50 respondents. A qualitative, content analysis approach was taken to analyse the data. First, responses were coded using initial categories, and then they were clustered into emerging themes.

## 3. Results 

The results include both qualitative and quantitative data analyses and are presented within the following six broad themes: children’s level of functioning, children’s academic skills reported by parents, home learning activities and resources, parental expectations, parents’ and children’s attitudes towards literacy and mathematics, and use of technology to support home learning activities. The final section presents the correlation analyses.

### 3.1. Children’s Level of Functioning 

Table 2 reports the descriptive statistics for VABS-II and children’s chronological age. There were no significant differences in age between the DS group and the WS group, U = 451.5; *p* = 0.627, with children across the groups aged between 4.08 and 11.58 years (M = 95.95, SD = 26.86). In line with the populations cognitive profile, there was a significant difference between the two groups for the VABS-II expressive scale, U = 585.0; *p* = 0.011, with the DS group (M = 18.80, SD = 9.71) reporting lower scores than the WS group (M = 25.71, SD = 4.97). 

One parent reported that their child had a double diagnosis of WS and autism, one child with DS had an additional diagnosis of developmental dyscalculia, and one other child with DS was reported to have an additional diagnosis of cortical visual impairment. English was the primary language for 96% of the children, with one child speaking Romanian at home and one family not providing this information.

As it can be seen in Table 3, the majority of children were attending a mainstream school and received some form of additional support in the last academic year. There was no statistically significant association between child diagnosis and type of school, as assessed by Fisher’s exact test, *p* = 0.594. In line with their cognitive profile, a higher percentage of children in the DS group (60%) received visual support as compared with the children in the WS group (25%). Some respondents reported Lego therapy, play therapy, and art therapy as further additional support. Only one respondent reported receiving additional support for mathematics, and it was not the family whose child was diagnosed with developmental dyscalculia.

### 3.2. Children’s Academic Skills

**Figure 1:**
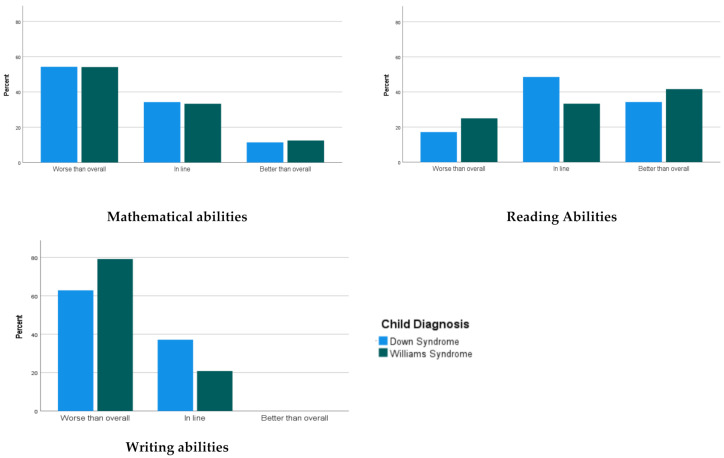
Parents were asked to rate whether their child’s academics abilities were better, in line, or worse than their overall abilities. The data represent the percentage of parents who agree with each statement.

When asked to compare their child’s overall abilities with their child’s academic abilities, and in particular mathematical, reading, and writing abilities, parents’ ratings were similar for both groups in all the academic domains, as indicated by a visual inspection of Figure 1. Most of the parents reported that writing and mathematics skills were a challenge for their child, with more than half of the parents in each group reporting that their child’s mathematical and writing skills were worse than their child’s overall abilities. On the other hand, more than 70% of the parents in both groups reported that their child’s reading abilities were either in line or better than their child’s overall abilities. 

When commenting on the challenges associated with reading abilities of their child, parents reported poor comprehension skills. For challenges related to writing, parents referred to “low muscle tone” or “hypermobile joints”, and several parents reported that their children were starting to learn to type instead of focusing on handwriting. Finally, when explaining mathematical difficulties of their children, most of the parents mentioned difficulties with “memory” and their child’s ability “to retain maths knowledge”. For example, one parent reported that their child “used to know 3 + 3 = 6, etc, but she doesn’t recall those as much now”. Some parents mentioned that their child found it difficult to “apply maths knowledge” and that they “struggle with abstract concepts” and “to apply [mathematical learning] and make links with prior learning”. 

### 3.3. Home Learning Activities and Resources

Parents were asked to provide information about the frequency of home learning activities. Table 4 shows the statistics computed separately for both formal and informal items, where possible, and for all the items included in each category.

A significant difference in the frequency of home learning activities between the DS group and the WS group was found only for the number skills category U = 201.0, *p* = 0.001, with parents of children with DS (M = 1.98, SD = 0.55) engaging more often in activities including counting and number recognition than parents of children with WS (M = 1.43, SD = 0.59). Overall, there was a statistically significant difference between the frequency of occurrence of home learning activities χ^2^(5) = 145.926, *p* < 0.0005. The post hoc analyses with Wilcoxon signed-rank test were conducted with a Bonferroni correction applied, resulting in a significance level set up at *p* = 0.003. Statistically significant differences were found between the frequency of occurrence of literacy activities and all the other categories, except for everyday life activities (z values between 5.932 and 6.487, all *p*-values < 0.0005) and between the frequency of everyday life activities and the occurrence of activities included in all the other categories, except for literacy (z values between 5.762 and 6.561, all *p*-values < 0.0005). No significant difference was found between the frequency of the three mathematics-based activities. The same pattern was found when analyses were run separately for the two groups. 

When comparing the frequency of formal and informal activities, a significant difference was found with respect to the number skills category in both the DS group (*z* = −2.519, *p* = 0.012) and the WS group (*z* = −3.650, *p* < 0.0005), with participants in both groups performing informal activities (DS group, M = 2.15, SD = 0.52 and WS group, M = 1.82, SD = 0.84) more often than formal activities (DS group, M = 1.85, SD = 0.79 and WS group, M = 1.01, SD = 0.64). 

In their comments, parents reported a wide range of different mathematics-based activities such as “count and sort toys”, “playing shops using pretend money”, “chant in 5s”, “read house numbers and bus stop adverts”, and having “discussions over dinner about maths problems”. Moreover, qualitative analysis of the comments confirmed that there were several difficulties, on both the child’s and the parent’s side, which affected the occurrence of mathematics-based activities at home. The difficulty reported the most was the lack of child’s motivation, both in terms of lack of interest and lack of attention. One parent reported “I find that after school my child is tired and has limited tolerance for attending to further educational activities” and another parent said, “I try to involve him but he does not seem interested, and often prefers to read instead”. Another factor that emerged was parents’ “lack of time”, with some parents reporting their wish to be “more consistent” in engaging in these activities with their child.

### 3.4. Parents’ Expectations

No significant differences between the DS and WS groups were found with respect to parents’ expectations towards their child’s academic abilities at the end of primary school, (U values between 316.5 and 454.5, all *p*-values > 0.110). As such, all further analyses were collapsed by group.

Table 5 shows that the average ES scores ranged from a minimum of 6.94 (arithmetic skills) to a maximum of 8.38 (number skills). Parents’ expectations for the control items were quite low (M = 4.03, SD = 2.47). This was expected, since the items included in the control category involved skills that are targeted in later academic years than the benchmark and skills that children are not exposed to during primary school. Despite having overall high expectations for their child’s academic abilities, one parent reported that “[…] she’s in her final year at primary [school]. I would have hoped she’d have achieved more of these as I would have had higher expectations if she was just starting school”. A statistically significant difference between parents’ expectations scores in the different categories was found: χ^2^(5) = 66.556, *p* < 0.0005. The post hoc analyses with Wilcoxon signed-rank test were conducted with a Bonferroni correction applied, resulting in a significance level set up at *p* = 0.003. Statistically significant differences were found between parents’ expectations towards number skills and all the other categories, except for literacy (z values between −6.027 and −3.171, all *p*-values < 0.002), between parents’ expectations towards arithmetic and all the other categories (z values between 3.282 and 6.027, all *p*-values < 0.001), and between parents’ expectations towards literacy and broader mathematical skills (*z* = 4.281, *p* < 0.0005). When commenting on their expectations for their child’s academic abilities, most of the parents stressed the importance of functional skills, with one parent reporting “I want her to have life skills, to be able to live and maybe work independently, to handle money day to day and to be able to budget or understand which product to buy”, and some parents referred to the importance of their child receiving “the right support, encouragement and positive praise”.

### 3.5. Parents’ and Children’s Attitudes towards Literacy and Mathematics

No significant differences between the DS group and the WS group were found in parents’ attitudes towards literacy: U = 389.0, *p* = 0.509, parents’ attitudes towards mathematics: U = 394.5, *p* = 0.682, and in the children’s reported interests towards mathematics: U = 329.5, *p* = 0.404. As such, all further analyses were collapsed by group.

As it can be seen in Table 6, parents reported significantly more positive attitude scores towards literacy (M = 4.79, SD = 0.61) as compared with mathematics (M = 4.34, SD = 0.86): *z* = 4.648, *p* < 0.0005. One parent reported “I know that some of my reluctance to tackle maths with my daughter is my own poor experience of maths at school […] and people with Down’s syndrome find maths difficult so perhaps I’ve been a bit defeatist”.

Overall, a significant difference was found between the average score of a child’s attitude towards mathematics and the parent’s attitude towards mathematics: *z* = 6.106, *p* < 0.0005, with the child’s attitude (M = 2.68, SD = 0.95) being lower than the corresponding parent’s attitude (M = 4.34, SD = 0.86). Three respondents reported all the items presented as not appropriate. Parents’ comments on their child’s attitudes towards mathematics were mixed. A few parents reported that their child “loves numbers and maths” and that their child “always chooses to do her maths homework first”. However, the majority of the parents reported that their children found mathematics “very hard” and reported different levels of engagement with the subject. One parent reported that their child “actively resists it”, one parent said that their child “wouldn’t choose to do math homework, but is okay about it with encouragement”, while another parent commented that their child often “enjoys maths more than she thinks she is going to”.

### 3.6. Use of Technology

Fifty-seven parents out of the 59 participants in the study sample reported that their child used technology at home. The results reported in this section only included data from these 57 participants. 

Fifty-two parents (91%) reported that their child had access to technology (e.g., tablets, computers, and smartphones) on a daily basis (Table 7), and 72% of parents reported that their children owned their own iPad or tablet. In their open-ended comments, most of the parents recognised the benefits of the use of technology and described it as an “essential tool of daily life”, with one parent reporting that their child “has a strength using technology and this should be maximised” and another parent observing that their child “seem [s] to learn a lot by what she watched”. It appeared that technology was mostly used to access videos and television programmes, while it was not used as often for playing videogames, making video calls, and reading e-books.

When comparing the frequency of watching literacy- and mathematics-based educational programmes, it was found that the frequency of watching literacy-based TV programme (M = 1.86, SD = 1.25) was higher than the average frequency of watching mathematics-based TV programme (M = 1.25, SD = 1.09) and that the difference in the scores was statistically significant, z = 4.046, *p* < 0.0005.

Furthermore, when analysing the data related to the use of mathematics-related apps (Table 8), the majority of parents were found to be using apps that target counting (63%), matching (53%), and number recognition (49%). However, at least half of the parents reported that their children were not using apps targeting arithmetic operations, work with number lines, and digital puzzles.

When commenting on the use of educational apps, parents reported their child’s high level of engagement, with one parent reporting that their child “loves the educational apps and will practice and practice things in a way he won’t do with me as he doesn’t like to make public mistakes”. Another parent observed that “through the use of iPad apps/websites her numeracy has made massive strides forward in the last 6 months”.

Finally, we investigated parental concerns around the use of technology (Table 9). More than one third of the parents (37%) reported that they were not concerned about their child’s use of technology. The remaining parents reported that their main concerns were related to the time spent in front of the screen and to the content of the applications not being appropriate. These results were confirmed by the comments of the parents that reported screen time rules and access limited to child-friendly applications to control the appropriateness of the content. When voicing their concerns, some parents mentioned the lack of knowledge around which applications are useful, and one parent reported that “there aren’t enough apps for kids with learning disabilities. There are some great apps out there […] but they aren’t adaptable, for example, in terms of speed of response”.

### 3.7. Correlations

To increase the power of statistical analyses and in view of the apparent homogeneity of the two groups, correlations were run on the whole sample rather than separately for the DS and WS groups.

Spearman’s correlations between the frequency of home learning activities and the VABS-II scores are shown in Table 10. 

Low negative correlations between the frequency of number-based activities and the scores for the VABS-II community scale, *rs*(59) = −0.283, *p* = 0.030 and fine motor skills scale, *rs*(59) = −0.257, *p* = 0.050, were found. A moderate to strong positive correlation between the frequency of arithmetic-based activities and the scores for all the VABS-II scales was observed; receptive *rs*(59) = 0.574, *p* < 0.0005; expressive *rs*(59) = 0.489, *p* < 0.0005; written: *rs*(59) = 0.563, *p* < 0.0005; community: *rs*(59) = 0.580, *p* < 0.0005; fine motor skills: *rs*(59) = 0.442, *p* < 0.0005. Moreover, moderate positive correlations between the frequency of literacy-based activities and the scores for the VABS-II expressive scale, *rs*(59) = 0.348, *p* = 0.007, and the community scale, *rs*(59) = 0.338, *p* = 0.009, were found. A low positive correlation was found between the frequency of literacy-based activities and the scores for the VABS-II fine motor skills scale, *rs*(59) = 0.293, *p* = 0.024.

Spearman’s correlations amongst the frequency of home learning activities, parents’ expectations, parents’ and children’s attitudes, and children’s chronological age are shown in Table 11. 

A moderate positive correlation between the frequency of home literacy-based activities and both everyday life activities: *rs*(59) = 0.528, *p* < 0.0005, and all the mathematics-based categories of home learning activities was observed, i.e., number skills: *rs*(59) = 0.359, *p* = 0.005; arithmetic: *rs*(59) = 0.464, *p* < 0.0005; and broader mathematical skills: *rs*(59) = 0.440, *p* < 0.0005. Moreover, we found a moderate correlation between the frequency of activities supporting arithmetic skills and broader mathematical skills; *rs*(59) = 0.477, *p* < 0.0005. In addition, a low to moderate positive correlation was found between the frequency of everyday life activities and the mathematics-based categories of home learning activities, i.e., number skills: *rs*(59) = 0.373, *p* = 0.004; arithmetic: *rs*(59) = 0.263, *p* = 0.044; broader mathematical skills: *rs*(59) = 0.479, *p* < 0.0005.

There were strong positive correlations between all the parental expectations categories. Furthermore, positive correlations between parental expectations and frequency for all literacy-based and mathematics-based activities were found, except for the frequency of activities supporting number skills. In particular, positive correlations between frequency of activity and corresponding expectations were found for all parental expectation categories except number skills, i.e., arithmetic: *rs*(59) = 0.570, *p* < 0.0005; broader mathematical skills *rs*(59) = 0.435, *p* = 0.001; literacy: *rs*(59) = 0.508, *p* < 0.0005; and everyday life skills: *rs*(59) = 0.280, *p* = 0.032.

There was a moderate positive correlation between parents’ attitudes towards literacy and parents’ attitudes towards mathematics: *rs*(59) = 0.357, *p* = 0.005, and frequency of arithmetic activities: *rs*(59) = 0.317, *p* = 0.015, and all the categories of parental expectations (number skills: *rs*(59) = 0.319, *p* = 0.014; arithmetic: *rs*(59) = 0.361, *p* = 0.005; broader mathematical skills *rs*(59) = 0.357, *p* = 0.005; and literacy: r(59) = 0.373, *p* = 0.004). Moreover, a weak positive correlation between parental attitudes towards mathematics and frequency of broad mathematical activities was observed *rs*(59) = 0.267, *p* = 0.041.

A low positive correlation between child’s attitudes towards mathematics and frequency of number skill activities: *rs*(56) = 0.285, *p* = 0.033 and literacy activities: *rs*(56) = 0.283, *p* = 0.034, was observed, and moderate positive correlations between child’s attitudes towards mathematics and frequency of activities targeting arithmetic skills: *rs*(56) = 0.350, *p* = 0.008, and parental attitude towards mathematics: *rs*(56) = 0.313, *p* < 0.019 were found.

Finally, low to moderate negative correlations were found between chronological age and frequency of activities targeting number skills: *rs*(59) = −0.323, *p* = 0.005, parental expectations towards their child’s arithmetic skills: *rs*(59) = −0.294, *p* = 0.024, and parental expectations towards their child’s everyday life skills: *rs*(59) = −0.269, *p* = 0.039. A moderate positive correlation was observed between chronological age and frequency of activities targeting arithmetic skills: *rs*(59) = 0.317, *p* = 0.005.

## 4. Discussion

This study was conducted to gain a detailed understanding of the home learning environment of primary school children with DS and primary school children with WS. The data were collected from 58 parents and one foster parent, predominantly across UK. This is the first study to explore and compare the home learning environment of children with DS and children with WS and to investigate whether, in these populations, the home learning environment varies to accommodate for the respective cognitive profiles. In doing so, this study explored the HNE of these populations for the first time.

A series of Mann–Whitney U tests showed that the home learning environment provided by parents of children with DS and children with WS was consistent across the two groups. In fact, for the most part, the reported frequency of activities, level of expectations, and parental attitudes did not differ significantly between the two groups. The only exception was that parents of children with DS provided mathematics-based activities that support counting and number recognition more often than parents of children with WS (Table 4). This finding could be explained by the well-known difficulties of children with DS with counting [36,37]. In addition, considering that, although most individuals with WS have good knowledge of counting names, their understanding of numbers and how they relate to each other is often poor [38], our results suggest that parents of children with WS recognise the difficulties that their children have with mathematics but might underestimate the difficulties that they have specifically with counting. Further studies should investigate if this is the case. 

An examination of correlations (Table 10) showed that children scoring lower on the VABS-II were frequently involved in activities supporting number skills, while children with higher levels of functioning were frequently involved in arithmetic- and literacy-based activities. Taken together, these findings suggest that the home learning environment changed on the basis of the child’s strengths and weaknesses, but that it was not syndrome specific.

In agreement with studies in TD populations [17,18] and with our predictions, literacy-based activities occurred significantly more frequently than mathematics-based activities. The same pattern was observed in the content of educational TV programmes children were exposed to, with children watching literacy-based TV programmes more often than mathematics-based TV programmes. This finding showed that the home learning environment of children with DS and children with WS is characterized by a rich HLE as compared with the HNE and it might be explained by the significantly more positive attitudes that parents reported towards literacy as compared with mathematics.

For both groups, no significant difference between the frequency of the three mathematics-based categories of activities was found. This finding showed that parents of children with DS and children with WS provided a varied HNE including activities supporting counting and number recognition as well as more advanced mathematical skills, such as arithmetic and numeracy skills. This finding is in contrast with previous studies in TD populations, which showed that counting was the most frequent mathematics-based activity occurring at home (e.g., Blevins-Knabe, Austin, Musun, Eddy and Jones [18]). This discrepancy might be explained by the different survey used to investigate the home learning environment or by the sampling technique, which might have led to the selection of participants interested in the HNE. Another explanation could be that the age range of the sample of this study might have hidden age-specific differences with regard to occurrences of different mathematics-based activities, given the negative correlation between the frequency of counting activities and a child’s age. Furthermore, we found that parents that offered more learning opportunities supporting arithmetic skills also engaged more frequently in activities supporting broader mathematical skills. This finding seems to suggest that parents that offered activities targeting more advanced mathematical skills, also provided a richer HNE that included activities that focused on other aspects of mathematics, such as numeracy and number foundations. 

In agreement with the findings reported by Blevins-Knabe, Austin, Musun, Eddy and Jones [18] in TD population, that showed that parents who engaged with their child more often in literacy-based activities offered mathematical activities more frequently, we found that parents that engaged more in literacy-based activities provided a more varied and richer home learning environment. The same pattern was observed for the everyday life activities, in that, parents that frequently engaged with their child in activities supporting their child’s academic skills, also engaged more in activities supporting other areas of their child’s development, such as social skills and level of independence. 

Our results showed that parents engaged with their child in both formal and informal literacy and number and arithmetic-based activities. The only statistically significant difference between the frequency of formal and informal activities was found for the number skills category, for both groups. This finding showed that parents were more familiar with informal mathematical activities supporting counting and digit recognition skills than with formal activities. Furthermore, the difference in frequency between informal number and arithmetic-based activities might suggest that parents struggled to incorporate mathematical components into daily activities to support arithmetic and tended to approach those tasks with the specific purpose of teaching mathematical skills. In agreement with our prediction, this was reflected by the apps used by the children, which mainly targeted counting, matching, and number recognition, with very few parents reporting using apps to support arithmetic skills.

In agreement with our predictions, parents had high expectations for their child’s literacy and number skills, but their expectations for their child’s arithmetic skills at the end of primary school were significantly lower as compared with the other categories. Parents of younger children had higher expectations for their child’s arithmetic abilities and everyday life skills as compared with parents of children approaching the end of primary school. Parents with high expectations tended to have high expectations overall, rather than for a specific category of their child’s academic abilities. Furthermore, parents that had higher expectations for their child were, in general, offering more frequent learning activities at home. These findings are in agreement with previous studies in TD population and applied to advanced mathematical activities [22] and also to literacy-based activities.

In contrast with our predictions, our findings neither support nor disagree with previous studies in TD populations [18], suggesting that the frequency with which parents involved their child in mathematical activities was positively related to their own attitudes towards mathematics. However, we found a weak positive correlation between parental attitudes towards mathematics and the frequency of broad mathematical activities. This finding seemed to suggest that parents with a more positive attitude towards mathematics are the parents who are more likely to take advantage of opportunities to incorporate numeracy components into the HNE.

Finally, our findings showed a positive correlation between child and parental attitudes towards mathematics and that, overall, children had more negative attitudes towards mathematics than their parents. Moreover, our findings showed that children with more positive attitudes towards mathematics tended to engage more often in both literacy and mathematics-based activities at home. 

There are some important limitations to consider when interpreting the results. First, our sample mainly consisted of highly educated white families, and this must be taken into consideration when interpreting findings related to the type and frequency of home learning activities or to parents’ attitudes. Second, because of the nature of the online survey, this study could only cover the population with internet access. Furthermore, the results might be affected by accuracy issues due to the use of self-reported data. Third, this study might be limited in the extent to which it is able to accurately operationalise the home learning environment. Studies integrating different measures of the home learning environment, such as naturalistic observation and interviews, would add weight to the literature. In addition, no measures of academic outcome were collected. Hence, the association between academic outcome and home learning environment could not be investigated. Finally, this study only considered shared activities between child and parent. Further investigation of the home learning environment could include shared activities involving other members of the family (e.g., siblings) and could investigate how the structural characteristics of the families influence the home learning environment in these populations.

## 5. Conclusions

Overall, our results indicate that various learning activities take place in the homes of primary school children with DS or with WS, with literacy-based activities occurring more often than mathematics-based activities. This might be explained by the significantly higher level of positive attitudes of parents towards literacy than towards mathematics. However, the HNE was varied and characterized by activities supporting different mathematical skills such as counting, arithmetic, and numeracy. Parents in both groups engaged with their child in both formal and informal literacy and mathematic activities, but informal activities occurred more often when supporting counting and number recognition skills. Moreover, our results show that parents that provide a rich home learning environment engage with their child in activities supporting their child’s broader development. 

In conclusion, our results suggest that the type and frequency of home learning activities changed on the basis of the child’s cognitive profile, showing an interaction between cognitive and environmental factors. Moreover, our findings show that the home learning environment of children with DS and children with WS were similar, with the exception of activities supporting counting, where parents of children with DS engaged in such activities more often than parents of children with WS. 

The implications of the present study are two-fold. On the one hand, the findings provide information that could inform the development of parental interventions aimed at improving parents’ levels of confidence towards mathematics and highlight opportunities to enrich the HNE and, subsequently, the home learning environment. On the other hand, the findings could be used by school staff and other professionals working with the child and the family to support consistency between different settings and to support the child’s development outside the home environment.

## Figures and Tables

**Table 1:** Sociodemographic characteristics of the participants.

	n	%
Gender		
Female	58	98
Male	1	2
Ethnicity		
White	53	90
Asiatic	3	5
Other	3	5
Highest level of education		
University degree	44	75
A level (or equivalent)	12	20
Vocational training	2	3
Missing	1	2
Highest level of mathematical education		
Doctoral level	1	2
Undergraduate level	7	12
Secondary school	48	81
Missing	3	5
Country		
UK	57	96
Ireland	1	2
Australia	1	2

Note: N = 59, n = number in subsample, % = percentage.

**Table 2:** Mean (M), standard deviation (SD), minimum (Min) and maximum (Max) value for chronological age in months (CA), and raw scores of each subscale of the VABS-II, by group.

	DS			WS			Mann–Whitney Test
	M (SD)	Min	Max	M (SD)	Min	Max	
VABS-II receptive	15.80 (5.35)	6.00	24.00	16.21 (5.12)	6.00	23.00	U = 436.5, *p* = 0.799
VABS-II expressive	18.80 (9.71)	1.00	30.00	25.71 (4.97)	9.00	30.00	U = 585.0, *p* = 0.011 (*)
VABS-II written	17.03 (8.07)	1.00	26.00	20.42 (6.28)	5.00	26.00	U = 521.0, *p* = 0.117
VABS-II community	21.23 (10.21)	5.00	42.00	24.96 (7.53)	9.00	38.00	U = 519.5, *p* = 0.124
VABS-II fine motor	25.46 (13.60)	5.00	49.00	27.46 (10.54)	7.00	43.00	U = 468.0, *p* = 0.459
CA (months)	94.80 (27.94)	50.00	142.00	97.63 (25.69)	54.00	140.00	U = 451.5, *p* = 0.627

Note: N (DS) = 35, N (WS) = 24, * = significance on Mann–Whitney test *p* < 0.05.

**Table 3:** Count (n) and percentage (%) of children attending different types of schooling and receiving additional support in the last academic year, by group.

	DS		WS	
	n	%	n	%
Type of schooling				
Mainstream school	27	77	17	71
SEN school	3	8	4	17
Mainstream school with SEN unit on site	2	6	3	12
Dual placement	1	3	0	0
Home educated	1	3	0	0
Nursery	1	3	0	0
Additional support (*)				
Speech and language therapy	33	94	18	75
Special educational needs support	27	77	23	96
Occupational therapy (sensory)	19	54	9	38
Visual supports	21	60	6	25
Extra reading help/phonics	21	60	15	63
Life skills teaching	7	20	8	33
Physiotherapy	4	11	9	38
Music therapy/music lessons	6	17	7	29

Note: N (DS) = 35, N (WS) = 24, n = number in subsample, % = percentage. * Total exceeds 100% because respondents were asked to check all that apply.

**Table 4:** Mean (M) and standard deviation (SD) of frequency of home learning activities, by category.

Category	Formal		Informal		Total		Mann–Whitney Test
	M	SD	M	SD	M	SD	
Number skills	1.50	0.83	2.00	0.69	1.75	0.62	U = 201.0, *p* = 0.001 (*)
Arithmetic skills	1.64	0.82	1.57	0.85	1.56	0.74	U = 499.0, *p* = 0.221
Broader mathematical skills	-	-	-	-	1.47	0.70	U = 411.0, *p* = 0.889
Literacy skills	2.50	0.52	2.50	0.50	2.47	0.46	U = 309.0, *p* = 0.084
Domain-general skills	-	-	-	-	1.58	0.59	U = 317.5, *p* = 0.113
Everyday life	-	-	-	-	2.48	0.43	U = 295.5, *p* = 0.052

Note: * Significance on Mann–Whitney test *p* < 0.05.

**Table 5:** Mean (M), standard deviation (SD), and ranges for the parents’ expectations scores, by category.

Category	M	SD	Min–Max	Mann–Whitney Test
Number skills	8.38	2.12	0.13–10.00	U = 454.5, *p* = 0.593
Arithmetic skills	6.94	2.52	0.38–10.00	U = 432.5, *p* = 0.847
Broader mathematical skills	7.60	2.20	0.75–10.00	U = 379.0, *p* = 0.527
Literacy skills	8.21	1.96	1.13–10.00	U = 379.5, *p* = 0.531
Domain-general skills	7.74	1.94	2.88–10.00	U = 332.0, *p* = 0.174
Everyday life	7.80	1.85	1.75–10.00	U = 316.5, *p* = 0.110
Control items	4.03	2.47	0.00–10.00	U = 324.0, *p* = 0.276

**Table 6:** Mean (M), standard deviation (SD), and range for the parent’s attitude (PA) and child’s attitude (ChA) scores.

Category	N	M	SD	Min–Max	Mann–Whitney Test
PA literacy	59	4.79	0.61	1.00–5.00	U = 389.0, *p* = 0.509
PA mathematics	59	4.34	0.86	1.00–5.00	U = 394.5, *p* = 0.682
ChA mathematics	56	2.68	0.95	1.00–5.00	U = 329.5, *p* = 0.404

**Table 7:** Frequency of technology use at home.

	Daily		Weekly		Monthly		Never	
	n	%	n	%	n	%	n	%
Has access to technology	52	91	3	5	2	4	0	0
Watches videos on YouTube	49	86	5	9	1	2	2	3
Watches literacy educational programmes	29	51	2	4	15	26	11	19
Uses drawing apps	14	25	9	16	10	17	24	42
Watches mathematical educational programmes	11	19	9	16	20	35	17	30
Plays video games	8	14	11	19	6	11	32	56
Makes video calls	8	14	12	21	9	16	28	49
Reads e-books	5	9	2	4	7	12	43	75

Note: N = 57, n = number in subsample, % = percentage.

**Table 8:** Use of mathematics-related apps at home in the last month.

	Yes		No		Not Appropriate	
	n	%	n	%	n	%
Counting apps	36	63	19	33	2	4
Size/matching apps	30	53	25	44	2	3
Number recognition apps	28	49	23	40	6	11
Addition and Subtraction games	23	40	28	49	6	11
Mathematics-related websites	22	39	33	58	2	3
Digital puzzle games	21	37	34	60	2	3
“Filling the gap” number games	18	32	35	61	4	7
Racing games	16	28	39	68	2	4

Note: N = 57, n = number in subsample, % = percentage.

**Table 9:** Parents’ concerns around their child’s use of technology.

	n	%
Time on screen	27	47
Not appropriate content	19	33
Accidental in-app purchase	14	25
Effectiveness of these apps	7	12
No concern	21	37

Note: N = 57, n = number in subsample, % = percentage. Total exceeds 100% because respondents were asked to check all that apply.

**Table 10:** Spearman’s correlation between the frequency of home learning activities (CF scores) and the VABS-II scores.

	CF—Number Skills	CF—Arithmetic	CF—Broader Maths	CF—Literacy
VABS-II receptive	− 0.203	0.574 **	0.157	0.226
VABS-II expressive	−0.189	0.489 **	0.192	0.348 **
VABS-II written	−0.195	0.563 **	0.280 *	0.255
VABS-II community	−0.283 *	0.580 **	0.317 *	0.338 **
VABS-II fine motor skills	−0.257 *	0.442 **	0.187	0.293 *

Note: n = 59, * *p* < 0.05, ** *p* < 0.01.

**Table 11:** Spearman’s correlation between the frequency of home learning activities (CF scores), parents’ expectations (ES scores), parents’ attitudes (PA), children’s attitudes (ChA), and chronological age of the children (CA).

	1	2	3	4	5	6	7	8	9	10	11	12	13
1. CF—Number skills	-												
2. CF—Arithmetic	0.179												
3. CF—Broader maths	0.181	0.477 **											
4. CF—Literacy	0.359 **	0.464 **	0.440 **										
5. CF—Everyday	0.373 **	0.263 *	0.479 **	0.528 **									
6. ES—Number skills	0.163	0.536 **	0.280 *	0.472 **	0.236								
7. ES—Arithmetic	0.226	0.570 **	0.339 **	0.440 **	0.284 *	0.762 **							
8. ES—Broader maths	0.134	0.455 **	0.435 **	0.494 **	0.380 **	0.760 **	0.807 **						
9. ES—Literacy	0.180	0.493 **	0.290 *	0.508 **	0.229	0.829 **	0.801 **	0.865 **					
10. ES—Everyday	0.311 *	0.365 **	0.240	0.310 *	0.280 *	0.544 **	0.619 **	0.673 **	0.670 **				
11. PA—Maths	0.001	0.238	0.267 *	0.198	0.263 *	0.181	0.096	0.214	0.121	0.113			
12. PA—Literacy	−0.108	0.317 *	0.159	0.118	0.084	0.319 *	0.361 **	0.357 **	0.373 **	0.300 *	0.357 **		
13. ChA Maths ^a^	0.285 *	0.350 **	0.153	0.283 *	0.251	0.340 *	0.481 **	0.290 *	0.288 *	0.432 **	0.313 *	0.094	
14. CA child	−0.323 *	0.317 *	0.155	−0.020	0.020	−0.134	−0.294 *	−0.189	−0.174	−0.269 *	0.253	0.060	−0.108

Note: n = 59, ^a^ n = 56, * *p* < 0.05, ** *p* < 0.01.

## Appendix A

**Table A1:** Parents presented of 36 home activities.

Category	Formal Items	Informal Items
Number skills	Using Numicon resources Using number flashcards Reading number story books that include numbers or counting	Using sticker reward charts Singing number songs together (e.g., five little monkeys) Counting during daily activities (e.g., counting the number of apples when cooking)
Arithmetic skills	Worksheets on addition and subtraction Using number activity books Doing maths homework	Elementary calculations during daily activities (e.g., “There are five apples in the fruit bowl. If I take one, how many apples are left?”) Playing board games that require elementary computations (e.g., with two dice) Recognising and finding half of a quantity, length, set of objects or shape (e.g., Can I have half of yours sweets?)
Broader mathematical skills	Handling and naming common 2D or 3D shapes Playing dominoes Using measuring tools such as a ruler when drawing or a scale when cooking Playing estimation games (e.g., Guess which one is more?) Talking about money when shopping Telling the time	
Literacy skills	Writing letters and/or words (e.g., writing birthday cards) Writing/typing your child’s name Reading books	Paying attention to letters and/or words during daily activities (e.g., cooking) Playing games that include writing and/or reading (e.g., fishbowl game) Learning new words during daily activities
Domain-general abilities that support mathematical skills	Drawing Playing memory games (e.g., shopping list) Playing jigsaw puzzles Doing connect the dots activities Creating patterns with concrete materials (e.g., creating a necklace alternating red and blue beads) Playing with building blocks such as Lego	
Everyday life	Playing sports Doing shopping Watching TV Listening to music Playing with toys/videogames together Cooking together	

Note: Only number skills, arithmetic skills and literacy skills include formal and informal items.
